# Nutrient patterns in children with Attention-Deficit/Hyperactivity Disorder: a case–control study

**DOI:** 10.1007/s00787-026-03002-w

**Published:** 2026-03-12

**Authors:** Sofia Pinto, Teresa Correia-de-Sá, Hernâni Gonçalves, Sofia Marques, Micaela Guardiano, Benedita Sampaio-Maia, Joana Ferreira-Gomes

**Affiliations:** 1 Departmento de Biomedicina, Faculdade de Medicina da Universidade Do Porto, 4200-319 Porto, Portugal; 2 i3S - Instituto de Investigação E Inovação Em Saúde, Universidade Do Porto, 4200-135 Porto, Portugal; 3 IBMC - Instituto de Biologia Molecular E Celular, Universidade Do Porto, 4200-135 Porto, Portugal; 4 Departamento Medicina da Comunidade, Informação E Decisão Em Saúde, MEDCIDS, Faculdade de Medicina da Universidade Do Porto, 4200-450 Porto, Portugal; 5 CINTESIS@RISE - Centro de Investigação Em Tecnologias E Serviços de Saúde, Faculdade de Medicina da Universidade Do Porto, 4200-450 Porto, Portugal; 6 Instituto de Psicologia E Ciências da Educação, Universidade Lusíada, 4100-348 Porto, Portugal; 7 CIPD - Centro de Investigação Em Psicologia Para O Desenvolvimento Positivo, Universidade Lusíada, 4100-348 Porto, Portugal; 8 Departamento de Pediatria, Unidade de Pediatria Do Neurodesenvolvimento, ULS São João, 4200-319 Porto, Portugal; 9 Faculdade de Medicina Dentária, Universidade Do Porto, 4200-393 Porto, Portugal

**Keywords:** Nutrient patterns, Nutritional psychiatry, Neurodevelopment, ADHD

## Abstract

Attention-Deficit/Hyperactivity Disorder (ADHD) is one of the most prevalent and highly impacting neurodevelopmental disorders worldwide. Beyond its complexity, it’s a childhood-onset condition influenced by both genetic and environmental factors. Among these, nutrition has emerged as a relevant modifiable factor, yet most studies have focused on isolated nutrients, overlooking dietary complex interplay. This study addresses that gap using a data-driven nutrient pattern approach. To identify nutrient patterns in children with ADHD and explore their association with behavioural symptoms. In this case–control study, 76 children aged 6–10 years (ADHD: *n* = 43, Neurotypical, *n* = 33) were included. Nutritional intake was assessed using 3-day food diaries and Principal Component Analysis (PCA) was applied to 24 total energy intakeadjusted dietary nutrients. Multivariable regression models assessed associations between nutrient patterns, individual nutrients, and ADHD-related symptoms measured by *Child Behaviour Questionnaire* (CBCL) and *Conners’ Parent Rating Scale-Revised: Short Form* (CPRS-R:S). Seven major nutrient patterns were identified, explaining 76.7% of the total variance. Although no group-level differences in overall patterns reached statistical significance, the Antioxidant–Mineral factor showed a near-significant lower score in the ADHD group (*p* = 0.077). Children with ADHD consumed significantly less Monounsaturated fats (MUFAs) (*p* = 0.003), Polyunsaturated fats (PUFAs) (*p* = 0.010), and vitamin C (*p* = 0.014). Vitamin A and selenium emerged as key nutrients associated with behavioral and emotional symptoms, and ADHD severity. Our findings suggest that children with ADHD may have lower intake of antioxidant-rich micronutrients and healthy fats, with implications for symptom severity. These results underscore the potential role for nutrition-based strategies in the clinical management of ADHD.

**Supplementary information:**

The online version contains supplementary material available at 10.1007/s00787-026-03002-w.

## Introduction

The World Health Organization (WHO) identifies neurodevelopmental disorders as a pressing public health challenge [1]. Among these, Attention-Deficit/Hyperactivity Disorder (ADHD) stands out as a prevalent global concern, marked by substantial healthcare and societal costs [2, 3]. This childhood-onset neurodevelopmental disorder is characterized by age-inappropriate levels of inattention, impulsivity, and hyperactivity, significantly affecting daily functioning and quality of life [4–6]. The multifactorial aetiology of ADHD is reflected in the heterogeneity of this disorder, as indicated by its diversity of psychiatric comorbidities, varied clinical profiles, and the wide range of structural and functional brain differences [7]. Twin studies have shown that ADHD is estimated to have a heritability of around 74% [8], but environmental factors also play a significant role [9, 10]. Evidence from studies on immune-related disorders, immune biomarkers, and genetic susceptibility suggests a role of immune dysregulation in ADHD [9, 10]. Additionally, prenatal inflammatory exposure has been proposed as a potential risk factor for the development of the disorder [11, 12].

Over the past decades, increasing notice has been directed towards the impact of nutrition on cognitive and behavioural functioning [13–15] with various nutrients and diet quality being linked to behavioural, cognitive, and affective functions, as well as to the prevalence of mental disorders [16]. While there is no evidence that diet alone is an aetiological factor, some studies suggest that certain dietary factors may influence ADHD symptoms and severity in some individuals [17, 18]. On one hand, children with ADHD may be at risk for a variety of nutrient deficiencies due to the attentional demands required to sit through a meal to obtain adequate levels of nutrient intake, as well as the appetite suppressant effects of stimulant medications [19]. On the other, genetic polymorphisms involved in micronutrient metabolism, such as variants in the methylenetetrahydrofolate reductase (MTHFR) gene, which have been reported in psychiatric disorders, may also contribute to an increased vulnerability to nutritional deficiencies in children with ADHD. In this line, recently the National Institute for Health and Care Excellence (NICE) guidelines have emphasized the value of a balanced diet and exercise for individuals with ADHD [20] and the Australian ADHD Professionals Association recommends addressing diet and physical activity levels in clinical practice [21].

As we delve into the existing literature, it becomes evident that the relationship between nutrient intake and ADHD is more complex than it seems, being influenced by genetic predispositions, other environmental factors, and individual variations [8, 22]. A case–control study found that children with ADHD presented lower intakes of protein, vitamins B1, B2, C, zinc and calcium [23]. Additionally, children with ADHD often exhibit diminished plasma concentrations of important brain function trace elements such as zinc, copper, iron, magnesium, and selenium [24–28]. While specific nutrient-ADHD associations have been explored, studies on nutrient patterns in ADHD remain limited. Dietary patterns, including nutrient patterns, represent an aggregate variable that is derived from special statistical methods, which summarizes nutrient intake to create a meaningful overall representation of a large and complex set of interrelated diet factors [29]. Rather than considering individual nutrients, nutrient patterns conceptually represent a broader picture of nutritional intake and may thus be more predictive of disease risk [29]. As so, this study aims to analyse nutrient patterns in ADHD children and to explore the intricate relationship between these patterns and ADHD symptomatology.

## Methods

The present study was developed in the ongoing M2Child project. All participants were invited to a one-time visit to the Medical Investigation Centre of the Faculty of Medicine of the University of Porto, Portugal (CIM-FMUP) from 2021 until now. In each visit, a thorough clinical and psychological evaluation was carried out, including performance tests, physical assessments, and questionnaires completion. The necessary sample size was calculated in G power [30] assuming an alpha of 0.05, a power of 0.8 and an effect size of 0.65, with a final value of 76 participants divided into two groups: ADHD and Neurotypical (NT). Participants were recruited from public hospitals, private clinics, and the community. Exclusion criteria included children diagnosed with autism spectrum disorder (ASD) as a primary diagnosis, intellectual disability, epilepsy, other brain disorders, or known genetic disorders.

This case–control study was approved by the Ethics Committee of University Hospital Centre of São João (CHUSJ; reference 318/2020) and conducted in accordance with the Declaration of Helsinki (1975, revised 2000). Written informed consent was obtained from parents/guardians, and verbal assent from children. Participation was voluntary and participants could withdraw at any time. Anonymity and data confidentiality were ensured throughout the study.

### Participants’ recruitment, (neuro)psychological evaluation, and anthropometric assessment

A total of 117 children, aged between 6 and 10 years, were recruited and evaluated. The diagnosis of ADHD was established according to clinical criteria of the Diagnostic and Statistical Manual of Mental Disorders – 5th Edition (DSM-5), following clinical evaluation by qualified professionals [5]. Afterwards, standardized assessment tools, including the Conners’ Parent Rating Scale-Revised: Short Form (CPRS-R:S) [31] and the Child Behaviour Checklist (CBCL), were administered to parents to evaluate participants’ behavioral and emotional problems. Participants were categorised according to cutoffs for normal, borderline, and clinical ranges based on T-score profiles for school-age children. For CBCL scoring, T-scores > 50 and ≤ 64 fell within the normal range, T-scores ≥ 65 and ≤ 70 fell within the borderline range, and T-scores ≥ 71 were within the clinical range. For CPRS-R:S scoring, T-scores ≤ 55 were considered in the normal range, T-scores > 55 and ≤ 60 were considered borderline, and scores > 60 were categorised as atypical. The subscales for both CBCL and CPRS-R:S, and Cronbach’s alphas, are described in Supplementary Materials – Table S3.

**Fig. 1:**
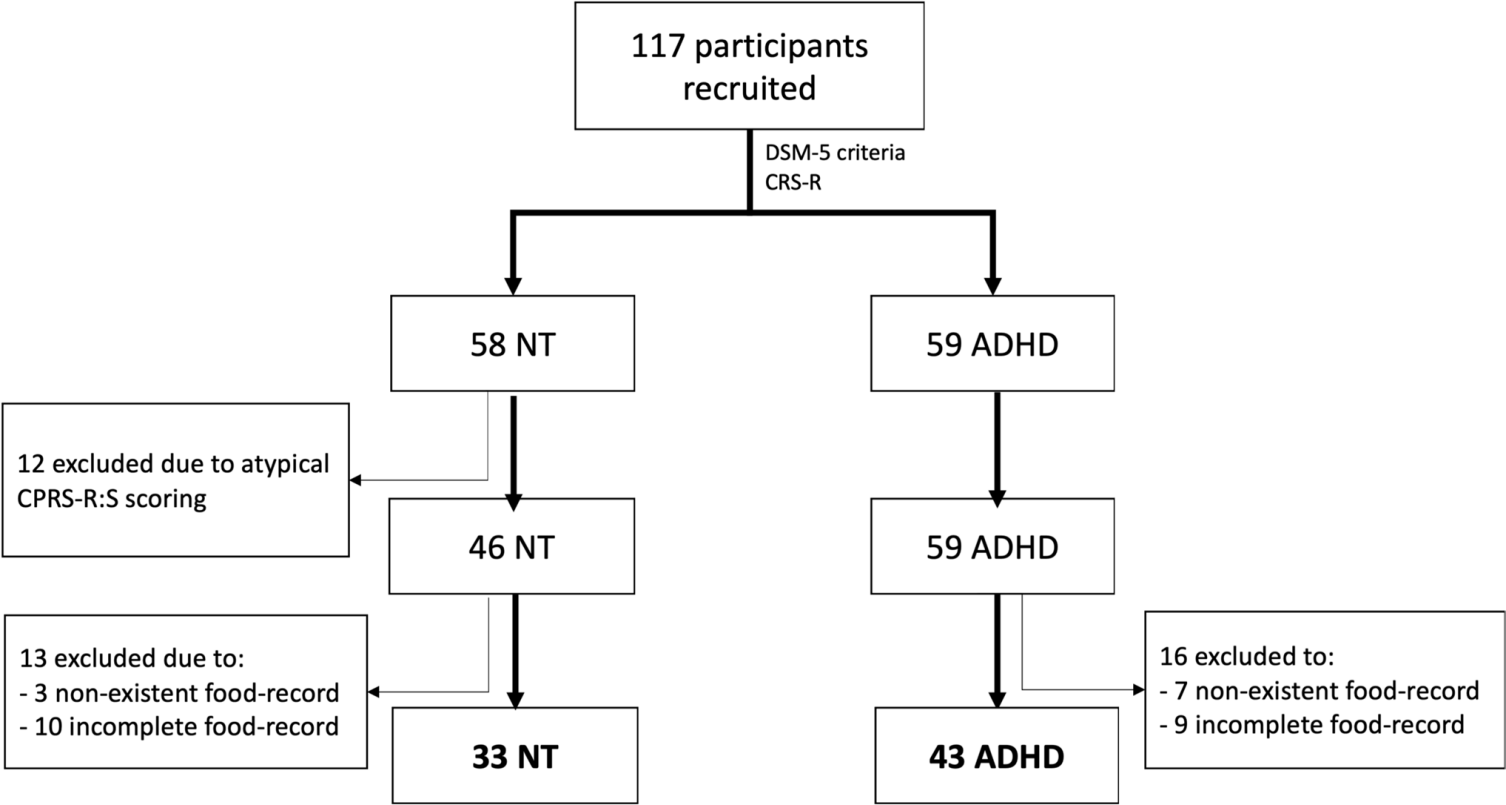
Flowchart of the sampling process for recruited Neurotypical (NT) and ADHD children

Twelve participants were excluded from the NT group due to atypical scoring in the CPRS-R:S. The final NT group therefore comprised children without a clinical diagnosis of ADHD or other neurodevelopmental disorders, including ASD, and with CPRS-R:S scores in the normal or borderline range only**.** Participants were further excluded based on completion of the food records: 13 (28%) children from the NT group and 16 (27%) from th ADHD group were excluded. The flowchart of the sampling process is presented in Fig. 1. Anthropometric measures, including weight and height for each participant, were obtained from the individual Child and Youth Health Bulletin. The measurements closest to the evaluation date were used, and data with more than six months’ difference were considered missing. These measures were then used to calculate body mass index (BMI) using the Quetelet Index (kg/m^2^) [32]. Physical activity levels, evaluated by parents’ report, were transformed into a categorical variable (practice/no practice) according to the practice of physical activity beyond mandatory school classes.

### Nutritional intake quantification

As part of their assessment, the parents of all children were requested to fill a 3-day consecutive food diary, including 2 weekdays and 1 weekend day, complemented with photographs of the children’s meals. This method was selected, as previous studies have demonstrated that this approach provides a representative estimate of habitual dietary intake while minimizing participant burden and risk of non-compliance [33]. The data obtained from these records were uploaded to the ESHA’s Food Processor® Nutrition Analysis software, version 11.15.49, which calculates macro- and micronutrient composition based on standardised food composition databases. Within this analysis, the following parameters were assessed: energy value, carbohydrates, added/free sugars, proteins, lipids, omega-3 and omega-6 fatty acids, dietary fibre, vitamins, and minerals.

### Statistical analysis

Initially, a descriptive analysis of the variables was carried out. Normally distributed continuous variables were described as the mean ± standard deviation value, while non-normally distributed variables were described by the median and percentiles 25 and 75 (P25-P75). The student’s *t*-test was used to compare groups of normally distributed data, otherwise, the *Mann–Whitney* test was applied. Categorical variables were presented as percentages and compared by the χ^2^-test or the *Fisher’*s exact test. Further, *Pearson* or *Spearman* correlation coefficients were used, according to the normality of the variables in study.

Multivariable regression models were used to assess the association between nutrients pattern scores and ADHD, and to adjust our models for potential confounders. In this context, nutrient pattern scores refer to the PCA-derived factor scores for each nutrient pattern, calculated for every participant as the weighted sum of standardized nutrient intakes using the corresponding factor loadings. Linear regression was performed for each nutrient, with nutrient intake as the dependent variable and group diagnosis as the independent variable. Energy-adjustment was done using Willet*’*s residual method [34]. In this method, the energy-adjusted intake estimate is the residual from a regression model in which total energy intake is the independent variable, and absolute nutrient intake is the dependent variable.

The considered significance level was 5% for all hypothesis tests and statistical analysis was performed using IBM SPSS Statistics software, version 29.

### Nutrient patterns analysis

Principal Component Analysis (PCA), an unsupervised machine learning algorithm, was used to derive major nutrient patterns. PCA does not separate groups (ADHD and NT) but rather analyses variance within the overall dataset, identifying patterns based solely on shared variability. Z-scores of each nutrient were calculated and then used in the PCA analysis. A step-by-step approach was utilized, where different rotation procedures were tested until the best fitted model (Kaiser–Meyer–Olkin Measure of Sampling Adequacy > 0.7 and Bartlett’s Test of Sphericity < 0.05) was achieved. The final model included a total of 24 nutrient z-scores. To identify the number of components to be retained three main criteria were used: (i) Eigenvalues > 1.0, (ii) elbow-point of the scree plot, and (iii) percentage of variance explained > 70% [35].

Initially, based on the visualization of the elbow-point of the scree plot, we identified two primary components. A two-dimensional (2D) scatter plot was computed to assess the distribution of these two components across the different diagnoses (ADHD vs. NT). Further analysis, guided by Eigenvalues greater than 1.0, unveiled seven significant components (Supplementary Fig. 1). These components were rotated by orthogonal transformation (Equamax procedure) to achieve a simpler structure with greater interpretability. Nutrients having higher absolute loadings were considered the primary contributors. The factor scores for each pattern and for each participant were determined by summing the intakes of each nutrient weighted by its factor loading [35]. These factor scores are hereafter referred to as nutrient pattern scores.

## Results

### Characterization of the study sample

The main characteristics of the study sample are presented in Table 1. Although no significant age differences were observed between groups, a significant disparity in gender distribution was noted (*p* = 0.022), with males comprising 76.7% of the ADHD group. All children from the NT group are classified in the upper-middle socioeconomic class, defined by the Graffar score, 20.9% and 2.3% of ADHD children are in the middle and lower-middle class, respectively (*p* = 0.006). No statistically significant differences between ADHD and NT children were found in any anthropometric measures and physical activity practice. Inter-group significant differences were also observed for use of ADHD medication (*p* < 0.001), with 75.6% of our ADHD sample being currently medicated. Moreover, the groups were significantly different according to the CPRS-R:S ADHD Index, (*p* < 0.001), being the ADHD group mostly atypical (88.4%), whereas the NT group was mostly normative (78.8%, *p* < 0.001). The ADHD group exhibited significantly higher values for all CBCL subscales (*p* < 0.05). In the subsequent analysis, variables with observed inter-group significant differences were included as confounding factors in the regression models.

**Table 1:** Description of sociodemographic factors, anthropometrical measures, ADHD-related measures, and child behaviours subscales of ADHD and NT groups

	ADHD (*n* = 43)	NT (*n* = 33)	*p*-value
Sociodemographic factors			
Age (years)^a^	8.5 ± 1.3	7.7 ± 1.7	0.068
Gender,* n* (%)			0.022
Male	33 (76.7)	17 (51.5)	
Female	10 (23.3)	16 (48.5)	
Socioeconomic level, *n* (%)			0.006
Lower-middle class	1 (2.3)	0 (0)	
Middle class	9 (20.9)	0 (0)	
Upper-middle class	33 (76.7)	33 (100)	
Anthropometrical measures			
Weight (kg)^a^	30.7 ± 8.0	27.7 ± 7.5	0.131
Height (m)^a^	1.3 ± 0.1	1.3 ± 0.1	0.079
BMI (kg/m^2^)^a^	16.9 ± 2.6	16.5 ± 2.9	0.231
Missing values, *n*	0	8	
Physical activity, *n* (%)			0.060
Not practice	7 (20.6)	1 (3.4)	
Practice	27 (79.4)	28 (96.6)	
Missing values, *n*	9	4	
ADHD-related measures			
ADHD Medication, *n* (%)			< 0.001
Yes	31 (75.6)	0 (0)	
No	10 (24.4)	33 (100)	
Missing values, *n*	2	0	
CPRS-R:S ADHD Index^1^, *n* (%)			< 0.001
Atypical	38 (88.4)	0 (0)	
Borderline	2 (4.7)	7 (21.2)	
Normative	3 (7.0)	26 (78.8)	
CBCL^2^ subscales			
Anxious/Depressed^b^	63 (57.0–68.8)	52 (50.0–62.0)	< 0.001
Withdrawn/Depressed^b^	66 (62.5–70.0)	54 (50.0–58.0)	< 0.001
Somatic Complaints^b^	57 (51.0–66.3)	53 (50.0–57.0)	0.013
Social Problems^b^	60 (54.5–65.0)	51 (50.0–53.0)	< 0.001
Thought Problems^b^	64 (54.5–50.8)	51 (50.0–56.0)	< 0.001
Attention Problems^b^	69 (64.0–72.5)	52 (50.0–57.0)	< 0.001
Rule-Breaking Behaviour^b^	60 (53.5–64.0)	51 (50.0–55.0)	< 0.001
Aggressive Behaviour^b^	65 (61.0–73.0)	52 (50.0–56-0)	< 0.001
Internalising problems ^a^	66.12 ± 8.58	52.84 ± 10.85	< 0.001
Externalising problems^b^	65 (60.0–71.0)	52 (41.0–56.3)	< 0.001

^1^Conners’ Parent Rating Scale-Revised: Short Form; ^2^Child Behaviour Questionnaire. Values are expressed in (a) mean ± standard deviation (SD) for normally distributed continuous variables and in (b) median (P25-P75) for non-normally distributed continuous variables

### Nutrient patterns identification

**Fig. 2:**
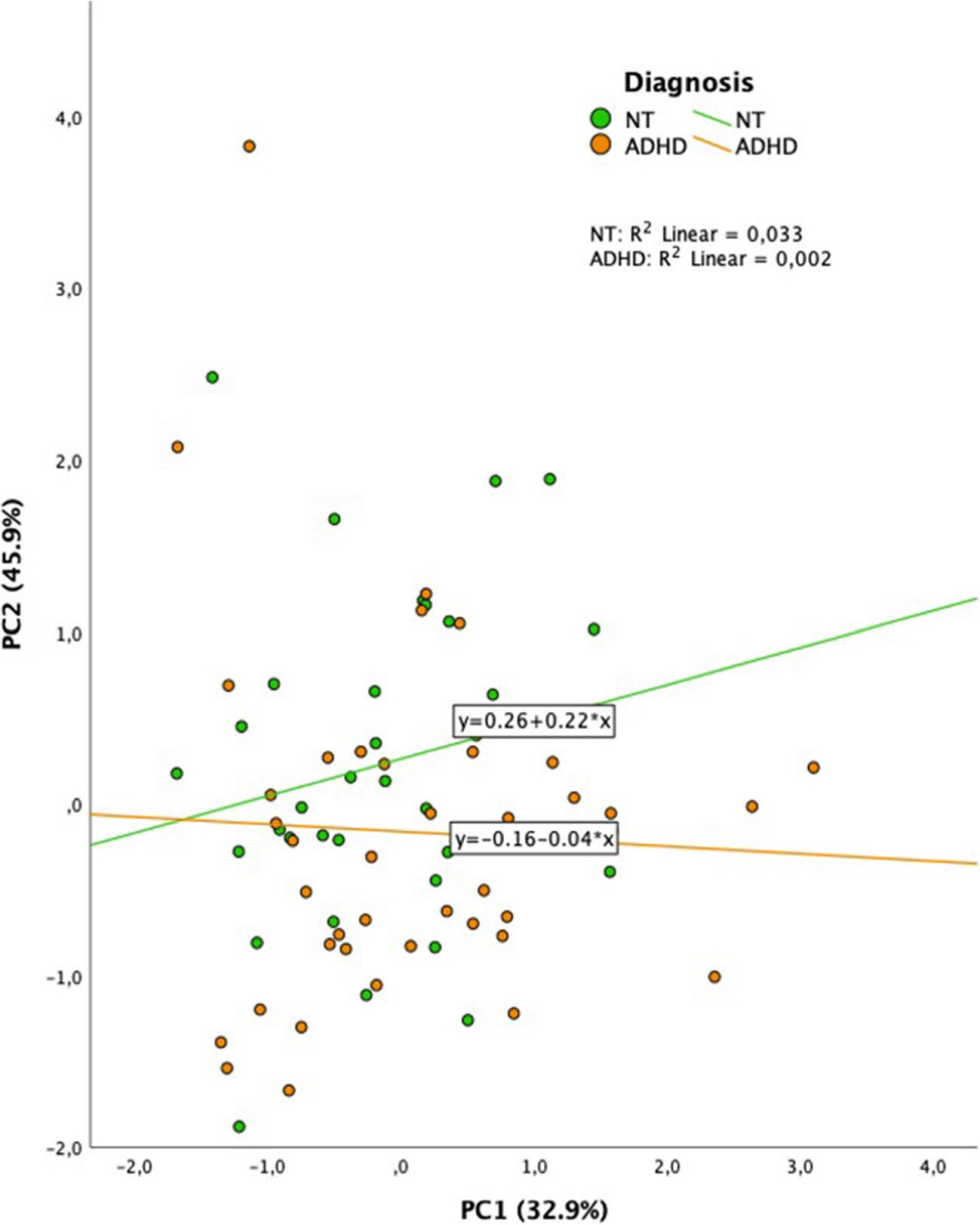
Two-dimensional scatter plot of principal component factor scores distinguished by diagnosis (ADHD vs. NT)

To explore underlying patterns in nutrient intake, we conducted a PCA, an unsupervised method that identifies components independently of diagnostic labels. When participants were subsequently grouped by diagnosis, the resulting 2D scatter plot of factor scores revealed distinct patterns between ADHD and NT children. Figure 2 depicts these nutrient patterns in a 2D plot where PC1 (X-axis) and PC2 (Y-axis) explained 32.9% and 13.1% of the total variance, respectively. T-test results indicated a marginally non-significant trend (*p* = 0.051), with the second principal component (PC2) showing the greatest distinction between ADHD and NT groups. This component (PC2) mainly includes micronutrients, whereas the PC1 mostly involves macronutrients. Within these, caloric and fat intake are the primary contributors to PC1, whereas folates and vitamin C mainly contribute to PC2 (Supplementary Table S1).

**Fig. 3:**
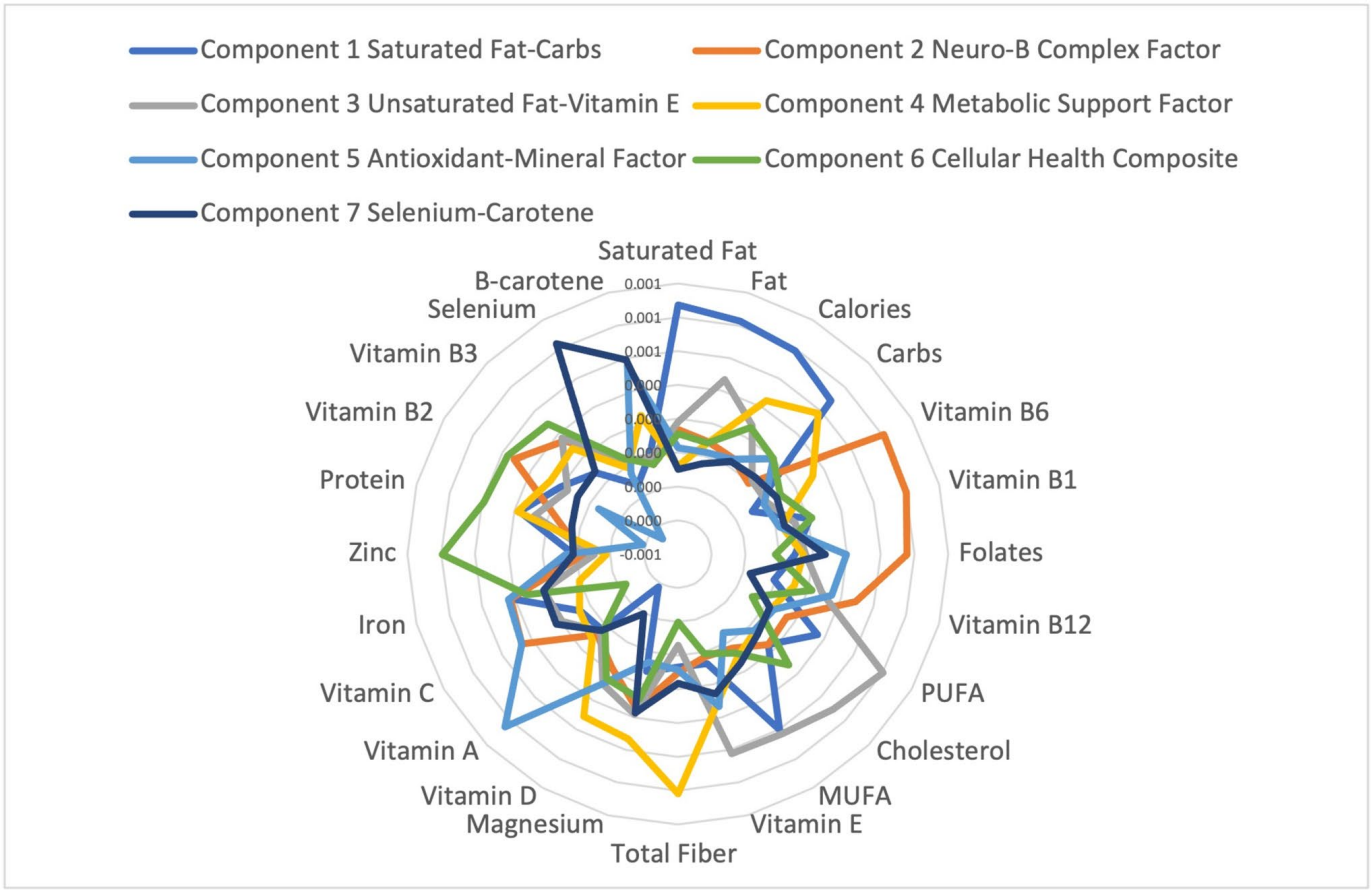
Radar graph representing nutrient pattern aggregation, distributed into seven principal components

Following the initial extraction of two principal components, we then proceeded with a more comprehensive analysis by retaining all components with Eigenvalues greater than 1, as per Kaiser’s criterion, resulting in the extraction of seven major components, that explained 76.7% of the total variance. The seven patterns extracted were named as: (1) Saturated Fat–Carbs, (2) Neuro-B Complex, (3) Unsaturated Fat-Vitamin E, (4) Metabolic Support Factor, (5) Antioxidant-Mineral, (6) Cellular Health Composite, and (7) Selenium-Carotene. The individual factor loadings of each component are further discriminated in Supplementary Table S2. The first nutrient pattern comprised z-scores of total fats, saturated fats, calories, and carbs, while the second pattern included B-complex vitamins such as B1, B6 and B12, and folates. The third pattern involved unsaturated fats (including mono- and poly- unsaturated), cholesterol and vitamin E. The fourth pattern encompasses metabolic support factors namely total fibre, magnesium, and vitamin D, while the fifth pattern includes vitamins with antioxidant capacity such as vitamins A and C, and iron. Finally, the sixth pattern comprises zinc, protein, and vitamins B2 and B3, and the seventh pattern, includes selenium and β-carotene. Figure 3 represents the aggregation of these seven principal components and the primary nutrient contributors to these components.

Overall, no statistically significant differences in nutrient patterns were found between the ADHD and NT groups (all p-value > 0.05). While median scores for most patterns were comparable across groups, a trend toward lower scores in the Antioxidant-Mineral Factor was observed in the ADHD group (*Mdn* = 1.63 vs. *Mdn* = −1.34; *p* = 0.077), approaching statistical significance.

### Nutritional intake quantification

To further interpret the underlying structure of the identified components, we examined the individual nutrients that showed meaningful contributions to each principal component, defined by factor loadings greater than 0.4. This threshold was used to identify the most strongly contributing variables within each component.

In the fully adjusted model, children with ADHD showed significantly lower intake of monounsaturated fats (MUFAs) and polyunsaturated fats (PUFAs), within the—Unsaturated Fat-Vitamin E pattern, (*p* = 0.003 and *p* = 0.010, respectively), and reduced vitamin C intake, within the Antioxidant-Mineral Factor (*p* = 0.014). While unadjusted models also indicated lower intake of vitamin A and selenium in the ADHD group, these associations were no longer significant after adjustment.

No other significant differences in nutrients intake were observed between groups, see Table 2.

**Table 2:** Descriptive statistics of nutrients intake in both groups, with univariable and multivariable linear regression models to test inter-group differences for each nutrient

Components	Nutrients	ADHD (*n* = 43)	NT (*n* = 33)	*p*-value^a^	*p*-value^b^
PC1	Energy (kcal)	1783 (1564–2153)	1776 (1573–1940)	0.317	0.195
	Carbohydrates, g (%TEV)	238.49 ± 54.7 (53.50%)	225.33 ± 46.70 (50.75%)	0.227	0.260
	Sat fat, g (%TEV)	21.22 ± 6.5 (10.71%)	19.68 ± 7.05 (9.98%)	0.107	0.218
	Fat, grams (%TEV)	62.43 ± 16.1 (31.51%)	59.57 ± 15.76 (30.19%)	0.207	0.575
PC2	Vit B1 (mg)	0.72 ± 0.3	0.74 ± 0.26	0.848	0.767
	Vit B6 (mg)	1.0 (0.7–1.2)	1.1 (0.8–1.2)	0.285	0.953
	Folates (μg)	170.11 ± 82.8	184.13 ± 65.05	0.553	0.404
	Vit B12 (μg)	2.8 (1.8–4.0)	2.7 (1.8–4.5)	0.954	0.118
PC3	MUFAs, g (%TEV)	18.22 ± 6.5 (9.20%)	18.28 ± 4.64 (9.26%)	0.932	0.003
	PUFAs, g (%TEV)	7.55 ± 2.9 (3.81%)	8.24 ± 3.38 (4.18%)	0.402	0.010
	Vit E α-tocoferol (mg)	3.76 ± 1.8	4.46 ± 2.15	0.075	0.078
	Cholesterol (mg)	199.9 (159.5–303.5)	250.2 (205.7–288.0)	0.061	0.129
PC4	Total fiber (g)	14.48 ± 5.6	14.34 ± 4.31	0.984	0.436
	Vit D (IU)	19.2 (4.1–130.8)	44.9 (7.9–129.6)	0.150	0.757
	Magnesium (mg)	191.05 ± 49.9	188.29 ± 41.53	0.976	0.060
PC5	Vit C (mg)	67.82 ± 42.4	85.81 ± 46.85	0.033	0.014
	Iron (mg)	7.61 ± 2.5	8.02 ± 2.14	0.953	0.530
	Vit A (RAE µg)	137.24 ± 147.2	199.54 ± 138.27	0.036	0.509
PC6	Vit B2 (mg)	1.16 ± 0.4	1.10 ± 0.35	0.402	0.392
	Vit B3 (mg)	21.9 (17.6–25.4)	20.3 (16.1–26.4)	0.656	0.471
	Protein, g (%TEV)	82.23 ± 20.2 (18.45%)	75.48 ± 13.69 (17%)	0.085	0.766
	Zinc (mg)	7.48 ± 10.6	5.77 ± 1.60	0.298	0.943
PC7	Selenium (μg)	33.4 (21.7–67.3)	55.0 (37.9–75.3)	0.027	0.976
	β-Carotene (µg)	1108.5 (383.0–1653.9)	1258.6 (655.4–2017.7)	0.238	0.374

^a^ Unadjusted model (without confounding variables in the model); ^b^ Model adjusted for energy intake (Willett’s residual method), gender, age, medication, BMI, physical activity and socioeconomic level

TEV: *Total Energy Value*. Values are expressed in mean ± standard deviation (SD) for normally distributed continuous variables and in median (P25-P75) for non-normally distributed continuous variables

### Nutrient-ADHD related symptoms

Table 3 presents the correlation coefficients between the nutritional parameters for which intergroup differences were found—MUFAs, PUFAs, vitamin C, vitamin A, and selenium—and ADHD-related measures assessed by the CPRS-R:S and CBCL scales. Higher levels of behavioural and emotional problems were associated with lower intakes of specific nutrients.

**Table 3:** Correlation coefficients between key nutrients and ADHD-related measures

		MUFAs^3^ (g)	PUFAs^4^ (g)	Vit A (µg)	Vit C (mg)	Sel (μg)
CPRS-R:S^1^	Cognitive Problems/Inattention	−0.052	0.037	−0.273^*^	−0.212	−0.104
	Hyperactivity/Impulsivity	−0.032	0.100	−0.258^*^	−0.270^*^	−0.157
	ADHD Index	−0.052	0.048	−0.323^**^	−0.282^*^	−0.167
CBCL^2^	Anxious/Depressed	0.024	−0.032	−0.191	−0.097	−0.124
	Withdrawn/Depressed	0.037	0.016	−0.200	−0.072	−0.220
	Somatic Complaints	−0.009	−0.051	−0.196	−0.197	−0.317^**^
	Social Problems	−0.047	−0.119	−0.238^*^	−0.116	−0.230^*^
	Thought Problems	−0.008	−0.012	−0.167	−0.185	−0.257^*^
	Attention Problems	0.031	0.073	−0.231^*^	−0.218	−0.137
	Rule-Breaking Behaviour	0.106	0.138	−0.251^*^	−0.206	−0.285^*^
	Aggressive Behaviour	0.091	0.118	−0.209	−0.176	−0.238^*^
	Internalizing Problems	−0.004	−0.054	−0.206	−0.124	−0.224
	Externalizing Problems	0.128	0.142	−0.258^*^	−0.182	−0.227^*^

^1^Conners’ Parent Rating Scale-Revised: Short Form; ^2^Child Behaviour Questionnaire*;* ^3^Monounsaturated fats; ^4^Polyunsaturated fats

^**^ Correlation is significant at the 0.01 level; * Correlation is significant at the 0.05 level

Lower intake of vitamin A was consistently associated with higher levels of inattention, observed in both CPRS-R:S and CBCL scales, alongside associations with increased hyperactivity/impulsivity, Social Problems, and Externalizing Problems such as Rule-Breaking Behaviour. Moreover, lower intake of vitamin A show negative correlation with the CPRS-R:S ADHD severity index.

Similarly, reduced vitamin C intake was associated with higher hyperactivity/impulsivity and the ADHD index. Regarding selenium, lower nutrient intake correlated with greater severity of somatic complaints, social and thought problems, as well as externalising behaviours such as rule-breaking and aggressive behaviour. PUFAs and MUFAs did not exhibit significant correlations with any of the symptoms studied.

## Discussion

The present study examined nutrient patterns in children with ADHD and neurotypical (NT) controls using Principal Component Analysis (PCA), a data-driven, unsupervised machine-learning technique that identifies patterns in complex datasets. We initially explored the data through a two-component solution to visualize the primary dietary variance and gain an intuitive understanding of the data distribution. In this initial phase of our PCA, we identified two primary components that together explained approximately 46% of the total variance in nutrient intake among participants. The first component (PC1) was predominantly composed of macronutrients, particularly calories and total fats, while the second component (PC2) was driven mainly by micronutrients, with folates and vitamin C being the primary contributors. Although PCA is an unsupervised method and does not consider diagnostic labels in the extraction of components, the bi-dimensional scatter plot of factor scores revealed emerging group-level differences when participants were subsequently categorized by diagnosis. Notably, PC2 showed a near-significant trend in distinguishing children with ADHD from neurotypical controls. This suggests that variation in micronutrient profiles may be particularly relevant in the context of ADHD. These findings highlight that micronutrient intake, as captured by PC2, may be particularly relevant when examining nutritional differences in ADHD studies.

This preliminary analysis provided a useful overview but did not fully capture the complexity of nutrients interrelationships. To further refine our analysis and identify more nuanced dietary structures, we extended the PCA to retain all components with Eigenvalues greater than 1, commonly accepted as meaningful in factor analysis. This resulted in the extraction of seven principal components, that reflected distinct nutrient patterns, which together explained 76.7% of the total variance in nutrient intake. They were (1) Saturated Fat–Carbs, (2) Neuro-B Complex, (3) Unsaturated Fat-Vitamin E, (4) Metabolic Support Factor, (5) Antioxidant-Mineral Factor, (6) Cellular Health Composite and (7) Selenium-Carotene. While no statistically significant intergroup differences were found for most patterns, certain trends emerged, particularly highlighting the fifth pattern, Antioxidant-Mineral Factor. When examining individual nutrients, children with ADHD had significantly lower intakes of monounsaturated (MUFAs) and polyunsaturated fatty acids (PUFAs), as well as vitamin C.

Our results share key similarities with previous studies that applied data-driven methods to nutrient pattern analysis in ADHD. Zhou et al. [36] conducted a case–control study in China, where they identified a “mineral–protein” pattern high in zinc, protein, selenium, riboflavin, and calcium that was associated with a 53% (95% CI, 29%–68%) decrease in the odds of ADHD. Another study by Gumma et al. in Egypt, [37] found that vitamin- and mineral-rich patterns reduced the odds of ADHD, whereas energy-dense macronutrient patterns increased it. Consistent with Zhou et al. [36], our Antioxidant–Mineral Factor, which includes vitamin A, vitamin C and iron, exhibited a trend towards lower scores in the ADHD group (p = 0.077), possibly reflecting that deficiencies in antioxidant-rich nutrients may contribute to the oxidative stress and neuroinflammation associated with ADHD [11, 38]. Similarly, reduced MUFAs and PUFAs intakes in the ADHD group align with findings from Gumma et al. [37], underscoring the role of healthy fats in neuronal function and anti-inflammatory pathways. Additionally, although no inter-group significant differences were found, vitamin A and selenium were the nutritional parameters most consistently correlated with behavioral and emotional domains, as well as ADHD symptom severity in the whole sample. This resonates with prior studies suggesting that selenium status may influence neurodevelopmental outcomes, as prenatal exposure to high maternal selenium levels has been associated with an increased risk of ADHD in the offspring (OR: 1.29; 95% CI: 1.04–1.56) [39]. The integration of both vitamin A and selenium in our symptom-specific correlation models offers a novel perspective and is consistent with prior findings linking nutrients with antioxidant properties and ADHD-related inflammation and oxidative stress [11, 38, 40, 41]. Also, in a physiological perspective, our findings align with prior research indicating that children with ADHD often have inadequate dietary intakes, which may influence their symptoms or be a consequence of attentional and behavioural challenges that might influence regular eating patterns [42, 43]. Moreover, the reduced intakes of MUFAs, PUFAs, and vitamin C in the ADHD group are in line with previous literature suggesting potential associations between these nutrients and cognitive and emotional processes [44, 45]. Other studies have proposed that PUFAs, particularly n-3 fatty acids, may contribute to neuronal membrane structure and neurotransmitter modulation [45], processes that are relevant to neurodevelopment and behaviour [44, 45]. Alterations in fatty acid status have been reported in individuals with ADHD, however, the extent to which these alterations contribute to symptom expression remains uncertain and warrants further investigation [46, 47]. Similarly, vitamin C has been reported to possess antioxidant properties and to act as a cofactor in redox-coupled reactions involved in neurotransmitter synthesis, including dopamine and norepinephrine [48]. While these mechanisms are biologically plausible, the extent to which lower vitamin C intake contributes to ADHD symptom expression remains uncertain. Nonetheless, reduced intake of antioxidant-related nutrients may potentially exacerbate oxidative or neurochemical imbalances that have been described in ADHD.

The divergence in findings with other previous studies, particularly the lack of strong associations between certain patterns (e.g., Selenium-Carotene) and ADHD in our study, may be attributed to methodological and contextual factors. Both these studies utilized Food Frequency Questionnaires (FFQs) to assess dietary intake, contrasting with our use of 3-day food diaries, which may provide a more precise snapshot of actual consumption but potentially miss habitual intake patterns over a longer period of time [33]. Moreover, unlike FFQs, food diaries are less prone to recall bias but may underrepresent sporadically consumed nutrients such as selenium, potentially explaining the absence of significant findings for this nutrient [49]. Additionally, differences in dietary habits between populations (e.g., Mediterranean versus Chinese or Egyptian diets) and sample sizes may also have influenced the detection of associations with certain nutrients. Our findings also suggest potential interactions between nutrient patterns and sociodemographic factors. In our sample, a higher prevalence of ADHD was found in boys and children from lower socioeconomic backgrounds which is in line with previous literature [50, 51]. However, these disparities may influence dietary habits, as limited access to nutrient-dense foods could exacerbate deficiencies. Another important consideration is the influence of medication, which was prevalent among the ADHD participants in our study. Stimulant medications are known to suppress appetite, potentially contributing to reductions in nutrient intake, highlighting the need for clinicians to monitor the nutritional status of medicated children, as this suppression may selectively reduce the intake of specific nutrient-dense foods [52].

Finally, one of the key strengths of our study is the in-depth exploration of the relationship between nutrients and ADHD, focusing on specific symptoms rather than a categorical ADHD phenotype. Because of the heterogeneity of clinical phenotypes within ADHD, the authors believe that by disaggregating it into its various symptoms, we can study more in depth the association between distinct profiles of ADHD and higher risk of nutritional deficiencies. Nevertheless, it is important to acknowledge several limitations when interpreting our findings. First, the sample size was calculated assuming a moderate-to-large effect size, yet several associations observed were small to moderate, suggesting limited power to detect subtle effects. However, such effect sizes are common in nutritional research and may still be relevant in multifactorial conditions such as ADHD. Additionally, while the study investigated most of the possible dietary cofactors prevalent in the target population, the total variance explained by the nutrient intake among participants was 76.7%, thus indicating the existence of other essential cofactors. Second, due to the observational translational study design, only possible associations between nutrient patterns and ADHD symptoms were found in a single timepoint, but no causality was established. Third, some variables, such as socioeconomic status, may have introduced potential bias due to significant differences between groups, however, this was accounted for by adjusting the multivariable regression models. Lastly, the latest Portuguese food composition tables were used as much as possible, but when in the absence of some food items, we used nutritionally similar food items or in, particular cases, the United States Department of Agriculture (USDA) food database. In conclusion, although no statistically significant differences were found in overall nutrient patterns between children with ADHD and neurotypical controls, specific trends emerged that merit further investigation. Notably, the Antioxidant–Mineral component, which includes vitamins with antioxidant properties such as vitamins A and C, showed a trend toward lower scores in the ADHD group. Further analysis revealed significantly lower intake of MUFAs, PUFAs, vitamin C, and selenium among children with ADHD. Importantly, vitamins A and C, as well as selenium, were among the nutrients most strongly correlated with behavioural and emotional symptomatology. These findings suggest that while overall nutrient patterns may not differ markedly between groups, deficiencies in specific nutrients may play a role in symptom severity. From a clinical perspective, the observed results are unlikely to translate into large, immediately noticeable changes in symptoms at an individual level. However, modest and consistent nutritional differences may still be clinically meaningful over time, particularly if they accumulate or interact with other biological and environmental risk factors.

Future studies should extend these findings by incorporating biochemical measures, such as blood-based nutrient, apply longitudinal designs to explore causality, and include larger samples to increase statistical power, confirm the robustness of the identified patterns, and account for socioeconomic, cultural, and medication-related influences. These insights may ultimately inform the development of targeted nutritional strategies as part of a broader, multidisciplinary approach to ADHD management.

## Contributions

JFG and BSM designed the research; SP, TCS and JFG conducted the research; SP, TCS and HG analyzed the data; SM, MG and JFG provided major clinical and scientific input and interpreted the clinical data; and SP, wrote the manuscript and had primary responsibility for final content. All authors read, edited, critically reviewed and approved the final manuscript.

## Supplementary information

Below is the link to the electronic supplementary material.

Supplementary file1 (DOCX 51 KB)

## Data Availability

No datasets were generated or analysed during the current study.

## Abbreviations

ADHD: Attention-deficit/Hyperactivity disorder
BMI: Body mass index
CBCL: Child behaviour questionnaire
CHUSJ: University hospital centre of São João
CIM-FMUP: Medical investigation centre of the faculty of medicine of university of Porto
CPRS-R:S: Conners’ parent rating scale-revised: short form
DSM-5: Diagnostic and statistical manual of mental disorders—5th edition
MUFAs: Monounsaturated fats
NICE: National institute for health and care excellence
NT: Neurotypical
PCA: Principal component analysis
PC: Principal component
PUFAs: Polyunsaturated fats
USDA: United States department of agriculture
WHO: World health organization
2D: Two-dimensional
