# Supplementary material for: Nutrient patterns in children with Attention-Deficit/Hyperactivity Disorder: a case–control study
Source: Eur Child Adolesc Psychiatry. 2026 Mar 12;35(6):1911–22. doi: 10.1007/s00787-026-03002-w (PMC13337956; doi:10.1007/s00787-026-03002-w)
Supplement: Supplementary file 1 — Supplementary file1 (DOCX 51 KB) [file 787_2026_3002_MOESM1_ESM.docx]

**Supplementary Materials**


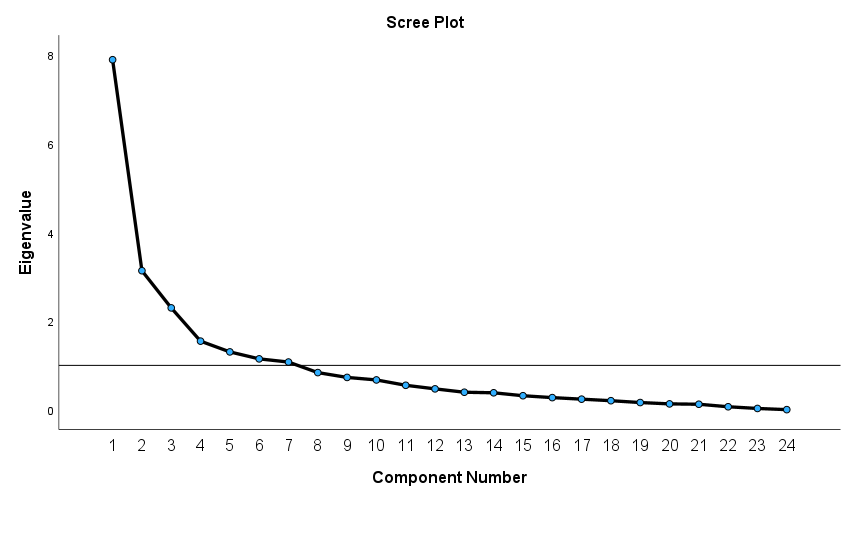


**Figure S1.** Scree plot of PCA showing the Eigenvalue threshold (cut-off = 1) used for component selection.

**Table S1.** Factor loadings of the initial two principal components: Macronutrient (PC1) and Micronutrient (PC2) patterns.

| Nutrients (z-scores) | PC1 | PC2 |
| --- | --- | --- |
| Calories | 0.895 | 0.149 |
| Total Fats | 0.879 | 0.016 |
| MUFAs^1^ | 0.809 | 0.079 |
| Protein | 0.793 | 0.001 |
| Saturated Fat | 0.763 | -0.081 |
| Carbs | 0.657 | 0.230 |
| PUFAs^2^ | 0.629 | 0.215 |
| Vitamin B3 | 0.617 | 0.127 |
| Cholesterol | 0.600 | 0.252 |
| Vitamin B2 | 0.566 | 0.405 |
| Zinc | 0.207 | -0.058 |
| Folates | 0.184 | 0.843 |
| Vitamin C | 0.033 | 0.673 |
| Vitamin B6 | 0.151 | 0.644 |
| Magnesium | 0.516 | 0.616 |
| Β-carotene | -0.071 | 0.602 |
| Vitamin A | -0.077 | 0.591 |
| Iron | 0.503 | 0.555 |
| Vitamin B12 | 0.239 | 0.530 |
| Vitamin B1 | 0.402 | 0.516 |
| Vitamin E | 0.361 | 0.503 |
| Vitamin D | 0.043 | 0.502 |
| Total Fiber | 0.109 | 0.443 |
| Selenium | -0.058 | 0.307 |

Extraction Method: Principal Component Analysis. Rotation Method: Equamax with Kaiser Normalization. ^1^Monounsaturated fats; ^2^Polyunsaturated fats*.*

**Table S2.** Factor loadings of all seven PCA-derived nutrient patterns after Equamax rotation.

| Nutrients  (z-scores) | 1 | 2 | 3 | 4 | 5 | 6 | 7 |
| --- | --- | --- | --- | --- | --- | --- | --- |
|  | Sat Fat-Carbs | Neuro-B Complex | Unsat Fats-Vitamin E | Metabolic Support | Antioxidant-Mineral | Cellular Health Composite | Selenium-  Carotene |
| Saturated Fat | 0.876 | 0.137 | 0.181 | -0.077 | 0.028 | 0.111 | -0.099 |
| Total Fats | 0.827 | 0.087 | 0.467 | 0.078 | 0.023 | 0.079 | -0.046 |
| Calories | 0.788 | 0.053 | 0.280 | 0.446 | 0.049 | 0.263 | 0.031 |
| Carbs | 0.682 | -0.005 | 0.019 | 0.579 | 0.200 | 0.199 | 0.049 |
| Vitamin B6 | -0.091 | 0.811 | 0.010 | 0.324 | -0.012 | 0.102 | 0.076 |
| Vitamin B1 | 0.211 | 0.803 | 0.118 | 0.038 | 0.023 | 0.222 | 0.056 |
| Folates | 0.093 | 0.756 | 0.158 | 0.146 | 0.399 | -0.021 | 0.275 |
| Vitamin B12 | -0.012 | 0.491 | 0.281 | 0.113 | 0.344 | 0.223 | -0.157 |
| PUFAs | 0.355 | 0.144 | 0.804 | 0.042 | 0.057 | -0.093 | 0.027 |
| Cholesterol | 0.156 | 0.160 | 0.699 | 0.044 | 0.041 | 0.331 | 0.069 |
| MUFAs | 0.602 | 0.044 | 0.632 | 0.104 | -0.065 | 0.076 | 0.143 |
| Vitamin E | 0.073 | 0.029 | 0.626 | 0.313 | 0.335 | 0.010 | 0.257 |
| Total Fiber | 0.071 | 0.103 | -0.061 | 0.821 | 0.087 | -0.199 | 0.164 |
| Magnesium | 0.119 | 0.347 | 0.384 | 0.532 | 0.064 | 0.286 | 0.374 |
| Vitamin D | -0.374 | 0.177 | 0.286 | 0.510 | 0.284 | 0.249 | -0.192 |
| Vitamin A | 0.031 | 0.076 | 0.048 | 0.119 | 0.846 | 0.011 | 0.040 |
| Vitamin C | 0.068 | 0.467 | 0.193 | 0.072 | 0.467 | -0.246 | 0.230 |
| Iron | 0.418 | 0.424 | 0.224 | 0.004 | 0.439 | 0.326 | 0.223 |
| Zinc | 0.016 | -0.045 | -0.104 | -0.176 | 0.066 | 0.793 | 0.018 |
| Protein | 0.369 | 0.125 | 0.279 | 0.383 | -0.386 | 0.588 | 0.050 |
| Vitamin B2 | 0.201 | 0.522 | 0.153 | 0.269 | -0.059 | 0.562 | 0.084 |
| Vitamin B3 | 0.082 | 0.346 | 0.373 | 0.284 | -0.472 | 0.489 | 0.093 |
| Selenium | -0.127 | 0.032 | 0.040 | -0.011 | -0.049 | 0.057 | 0.839 |
| Β-Carotene | 0.026 | -0.008 | -0.006 | 0.247 | 0.556 | -0.049 | 0.590 |
| % Variance | 32.874 | 13.060 | 9.570 | 6.447 | 5.437 | 4.783 | 4.483 |

Extraction Method: Principal Component Analysis. Rotation Method: Equamax with Kaiser Normalization. Grey shaded cells indicate the nutrients in the component with the highest absolute factorial load in each row line. ^1^Monounsaturated fats; ^2^Polyunsaturated fats.

**Table S3.** CBCL and CPRS-R:S reliability statistics including subscale constructs, item numbers, and Cronbach’s alpha coefficients.

| **Constructs** | **Items** | **No. of items** | **Alpha (⍺)** |
| --- | --- | --- | --- |
| **CBCL^1^** | | | |
| Anxious/Depressed | 14, 29, 30, 31, 32, 33, 35, 45, 50, 52, 71, 91, 112 | 13 | 0.825 |
| Withdrawn/Depressed | 5, 42, 65, 69, 75, 102, 103, 111 | 8 | 0.763 |
| Somatic Complaints | 47, 49, 51, 54, 56a, 56b, 56c, 56d, 56e, 56f, 56g | 11 | 0.830 |
| Social Problems | 11, 12, 25, 27, 34, 36, 38, 48, 62, 64, 79 | 11 | 0.855 |
| Thought Problems | 9, 18, 40, 46, 58, 59, 60, 66, 70, 76, 83, 84, 85, 92, 100 | 15 | 0.696 |
| Attention Problems | 1, 4, 8, 10, 13, 17, 41, 61, 78, 80 | 10 | 0.884 |
| Rule-Breaking Behaviour | 2, 26, 28, 39, 43, 63, 67, 72, 73, 81, 82, 90, 96, 99, 101, 105, 106 | 17 | 0.662 |
| Aggressive Behaviour | 3, 16, 19, 20, 21, 22, 23, 37, 57, 68, 86, 87, 88, 89, 94, 95, 97, 104 | 18 | 0.924 |
| Other Problems | 6, 7, 15, 24, 44, 53, 55, 56h, 74, 77, 93, 98, 107, 108, 109, 110, 113 | 17 | 0.551 |
| Internalising problems | 14, 29, 30, 31, 32, 33, 35, 45, 50, 52, 71, 91, 112, 5, 42, 65, 69, 75, 102, 103, 111, 47, 49, 51, 54, 56a, 56b, 56c, 56d, 56e, 56f, 56g | 32 | 0.900 |
| Externalising problems | 2, 26, 28, 39, 43, 63, 67, 72, 73, 81, 82, 90, 96, 99, 101, 105, 106, 3, 16, 19, 20, 21, 22, 23, 37, 57, 68, 86, 87, 88, 89, 94, 95, 97, 104 | 35 | 0.923 |
| Total problems | 11, 12, 25, 27, 34, 36, 38, 48, 62, 64, 79, 9, 18, 40, 46, 58, 59, 60, 66, 70, 76, 83, 84, 85, 92, 100, 1, 4, 8, 10, 13, 17, 41, 61, 78, 80, 6, 7, 15, 24, 44, 53, 55, 56h, 74, 77, 93, 98, 107, 108, 109, 110, 113 | 53 | 0.918 |
| **CPRS-R:S^2^** | | | |
| Cognitive problems/Inattention | 3, 8, 12, 17, 21 | 5 | 0.938 |
| Hyperactivity-Impulsivity | 4, 9, 14, 18, 22, 25, 26 | 7 | 0.940 |
| ADHD Index | 1, 5, 7, 10, 13, 15, 17, 19, 21, 23, 25, 27 | 12 | 0.954 |

^1^Child Behaviour Questionnaire; ^2^Conners’ Parent Rating Scale-Revised: Short Form.
